# *Legionella pneumophila*: A Microorganism with a Thousand Faces—From Environmental Complexity to Clinical and Technological Challenges

**DOI:** 10.3390/microorganisms14091886

**Published:** 2026-08-25

**Authors:** Teresa Maria Assunta Fasciana

**Affiliations:** 1 Legionella Reference Laboratory, University of Palermo, 90127 Palermo, Italy; teresa.fasciana@unipa.it; 2 Department of Health Promotion, Mother and Child Care, Internal Medicine and Medical Specialties, University of Palermo, 90127 Palermo, Italy

*Legionella pneumophila* epitomizes the concept of a microorganism with “a thousand faces”, reflecting its extraordinary ecological adaptability, genetic diversity, and multifaceted clinical relevance. As a ubiquitous environmental bacterium capable of colonizing a wide range of natural and engineered water systems, *L. pneumophila* represents a persistent challenge for public health. The studies included in this Special Issue highlight how this pathogen continuously adapts to changing environments, technologies, and host conditions, revealing an intricate network of interactions that shape its epidemiology and pathogenicity. The multifaceted nature of *L. pneumophila*, encompassing genetic diversity, genomic variation, virulence and host interactions, water-system colonization, domestic exposure, and emerging technologies, is summarized in Figure 1.

The diversity of *L. pneumophila* is particularly evident at the genomic and epidemiological levels. The epidemiology of *L. pneumophila* is characterized by a striking genetic diversity among clinical isolates. As reported by González-Rubio et al. [1], more than 130 sequence types circulate within human populations; however, only a limited number of dominant clones, such as ST1, ST23, ST20, and ST42, account for most clinical cases. This coexistence of extensive diversity with clonal dominance suggests that specific genotypes may possess enhanced fitness or transmissibility, although the relationship between genotype and virulence remains unclear.

These findings are consistent with clinical observations reported by Caputo et al. [2], who highlighted the complexity of community-acquired *Legionella pneumophila* pneumonia, emphasizing the role of host susceptibility, comorbidities, and environmental exposure in determining disease severity and clinical outcomes.

At the same time, the lack of a clear correlation between genotype and virulence, also highlighted by González-Rubio et al., underscores the complexity of predicting pathogenic potential. While certain clones are frequently associated with disease, rare or less common genotypes may also give rise to outbreaks, suggesting that virulence is shaped by a combination of microbial, environmental, and host-related factors [1,2,3].

Another defining “face” of *L. pneumophila* is its pervasive presence in anthropogenic water systems. Environmental investigations, such as those conducted by Nielsen et al. [4], reveal extremely high colonization rates in public infrastructures, including primary school hot water systems. Their findings demonstrate that nearly all investigated systems were colonized, with a substantial proportion exceeding recommended safety thresholds.

Importantly, as reported by Nielsen et al. [4], water temperature plays a critical role in modulating *Legionella* spp. proliferation, with higher temperatures associated with lower bacterial loads. However, this study also highlights that temperature alone cannot fully control contamination. System design, water stagnation, and reduced usage contribute to biofilm formation and bacterial persistence, emphasizing the multifactorial nature of *Legionella* spp. ecology [5].

This ecological adaptability is further reflected at the genomic level. As reported by Barigelli et al. [6], environmental strains exhibit a highly dynamic genome characterized by extensive recombination and horizontal gene transfer. This genomic plasticity translates into functional heterogeneity, with significant strain-dependent differences in cytotoxicity and intracellular replication. These findings reinforce the concept that *L. pneumophila* cannot be considered a uniform pathogen but rather a collection of strains with diverse virulence strategies.

The complexity of *Legionella* spp. epidemiology is further highlighted by advances in molecular diagnostics. As demonstrated by Paglione et al. [7], whole-genome sequencing has emerged as a powerful tool for high-resolution epidemiological analysis. In particular, WGS enables not only the identification of infection sources but also their exclusion, representing a critical advancement in outbreak investigation and infection control. This represents yet another “face” of *Legionella*: a pathogen whose epidemiological pathways can only be fully understood through cutting-edge genomic approaches.

Beyond large-scale infrastructures, *L. pneumophila* also manifests in less expected contexts, revealing additional facets of its adaptability. As reported by Reinares Ortiz et al. [8], domestic ultrasonic humidifiers can act as amplification niches for the bacterium, generating high bacterial loads and facilitating aerosol transmission. These findings challenge the traditional focus on large water systems and suggest that domestic environments may represent an underrecognized source of exposure, particularly for vulnerable individuals.

The emergence of new technologies introduces further complexity. As discussed by Brookes et al. [9], the increasing adoption of hot water heat pump systems driven by sustainability and decarbonization goals may inadvertently create conditions favorable to *L. pneumophila* growth. These systems often operate at lower temperatures, within the optimal range for bacterial proliferation, highlighting a critical tension between energy efficiency and microbiological safety.

Taken together, these studies illustrate how *L. pneumophila* expresses its “thousand faces” across multiple dimensions: genetic diversity, environmental persistence, technological adaptation, and clinical variability. Each of these aspects contributes to the overall challenge of controlling this pathogen in modern settings.

*L. pneumophila* stands as a model organism for understanding the complexity of environmental pathogens in a rapidly evolving world. Its ability to adapt to diverse ecological niches, combined with its genomic plasticity and interaction with human-made systems, makes it a persistent and multifaceted public health concern.

As highlighted in this Special Issue, addressing the challenge of legionellosis requires an integrated and multidisciplinary approach. Future strategies must balance technological innovation with microbiological safety, incorporate advanced genomic tools into routine surveillance, and expand the scope of investigation to include both traditional and emerging sources of exposure. Only by embracing the full spectrum of its “thousand faces” will it be possible to effectively understand and control *L. pneumophila* in the years to come.

## Figures and Tables

**Figure 1:**
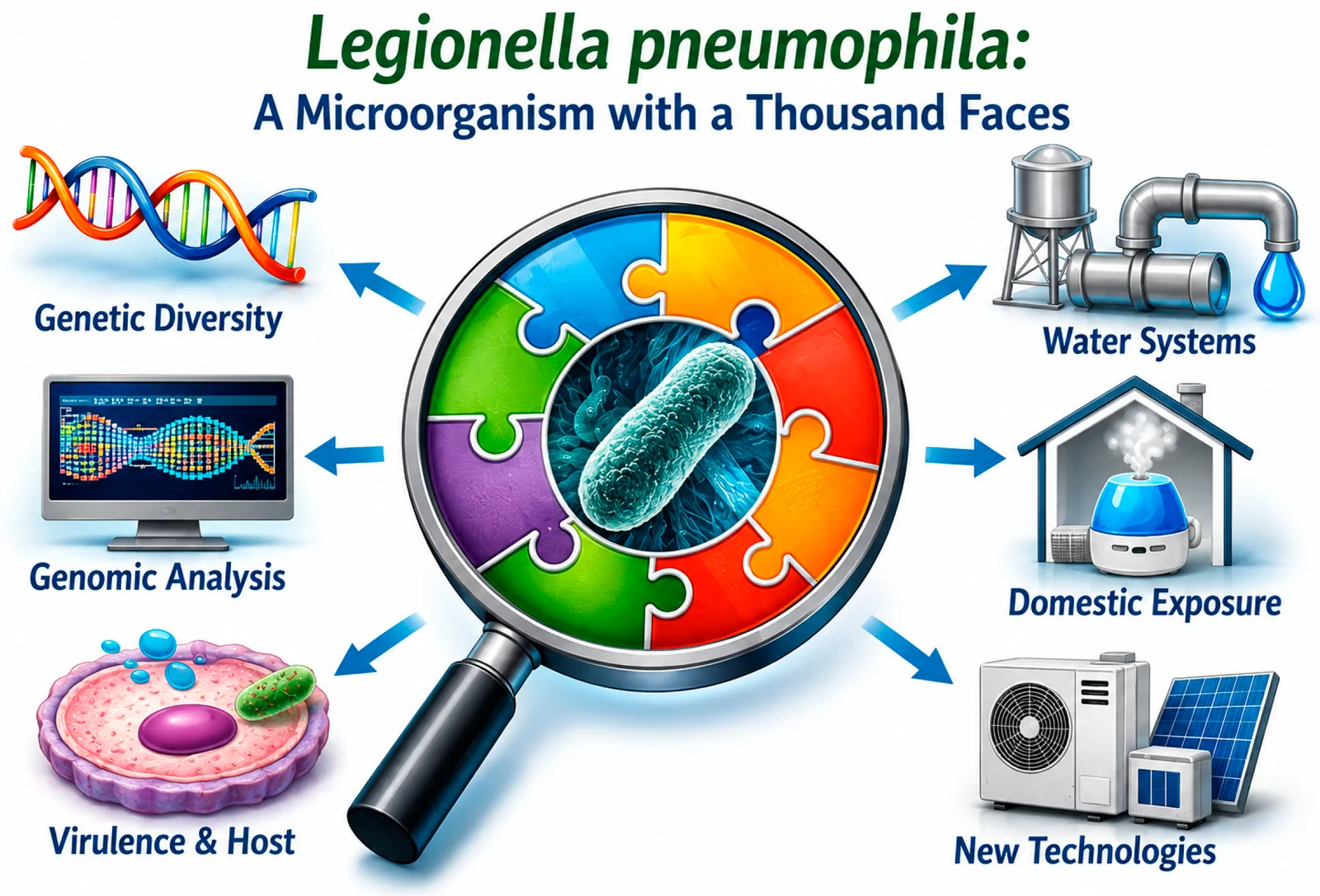
Schematic overview of the multifaceted nature of *Legionella pneumophila*, highlighting genetic diversity, genomic analysis, virulence and host interactions, water systems, domestic exposure, and emerging technologies.

## Data Availability

No new data were created or analyzed in this Editorial. Data sharing is not applicable to this article.
